# From Conventional Detection to Point-of-care Tests (POCT) Method for Pediatric Respiratory Infections Diagnosis: A Systematic Review

**DOI:** 10.34172/aim.33505

**Published:** 2025-02-01

**Authors:** Reza Azizian, Setareh Mamishi, Erfaneh Jafari, Mohammad Reza Mohammadi, Fereshteh Heidari Tajabadi, Babak Pourakbari

**Affiliations:** ^1^Pediatric Infectious Diseases Research Center (PIDRC), Tehran University of Medical Sciences, Tehran, Iran; ^2^Biomedical Innovation and Start-up Student Association (Biomino), Tehran University of Medical Sciences, Tehran, Iran; ^3^Department of Bacteriology, Faculty of Medical Sciences, Tarbiat Modares University, Tehran, Iran; ^4^Department of Microbiology, School of Medicine, Tehran University of Medical Sciences, Tehran, Iran

**Keywords:** Molecular diagnosis, Pediatric infections, POCT, Point-of-care testing, Respiratory infections

## Abstract

Bacterial respiratory infections pose significant health risks to children, particularly infants susceptible to upper respiratory tract infections (URTIs). The COVID-19 pandemic has further exacerbated the prevalence of these infections, with pathogens such as *Mycoplasma pneumoniae, Streptococcus pneumoniae, Legionella pneumophila, Staphylococcus aureus, Haemophilus influenzae,* and* Klebsiella species* commonly implicated in pediatric cases. The critical need for accurate and timely detection of these bacterial agents has highlighted the importance of advanced diagnostic techniques, including multiplex real-time PCR, in clinical practice. Multiplex real-time polymerase chain reaction (PCR) offers several advantages, including rapid results, high sensitivity, and specificity. By accelerating the diagnostic process, this approach enables early intervention and targeted treatment, ultimately improving patient outcomes. In addition to PCR technologies, rapid and point-of-care testing (POCT) play a crucial role in the prompt diagnosis of bacterial respiratory infections. These tests are designed to be user-friendly, sensitive, and deliver quick results, making them particularly valuable in urgent clinical settings. POCT tests are often categorized into two main groups: those aimed at determining the cause of infection and those focused on confirming the presence of specific pathogens. By utilizing POCT, healthcare providers can make rapid and informed treatment decisions, leading to more effective management of bacterial respiratory infections in children. As the medical community continues to explore innovative diagnostic approaches, the integration of molecular and rapid testing methods offers significant promise in the realm of bacterial respiratory infections. By adopting these cutting-edge technologies, healthcare professionals can enhance their ability to accurately diagnose these infections, tailor treatment strategies, and ultimately improve patient care.

## Introduction

 Molecular and conventional detection methods play a crucial role in the diagnosis and management of pediatric respiratory infections. Conventional methods, such as culture-based techniques, have long been the standard approach for identifying pathogens causing respiratory infections in children. However, these methods are time-consuming and may lack sensitivity, particularly for fastidious organisms.^1^ In contrast, molecular methods, including polymerase chain reaction (PCR) and nucleic acid amplification tests, offer rapid and accurate detection of a wide range of respiratory pathogens in pediatric patients.^2^

 These molecular techniques have revolutionized the field of diagnostic microbiology by providing sensitive and specific identification of viruses, bacteria, and fungi causing respiratory infections. The rapid and accurate diagnosis facilitated by molecular methods allows for appropriate and timely treatment, reduces unnecessary antibiotic use, and aids in infection control measures to prevent the spread of pathogens within healthcare settings.^3^

 The increasing prevalence of antibiotic-resistant bacteria has further underscored the importance of accurate and rapid diagnostic methods. More than 70% of uncertain diagnoses and prescriptions of antibiotics contribute to the rising antibacterial resistance in children.^4^ While clinical examination may suffice for diagnosing upper respiratory tract infections (URTIs), lower respiratory tract infections often present diagnostic challenges, necessitating more advanced techniques.^5^

 The effectiveness of microbiological tests is influenced by various factors, including the origin of the sample, the concentrations and pathogenic potential of different organisms, and the impact of previous antibiotic therapy.^6,7^ Common identification methods include X-ray imaging, culturing, gram staining, and biochemical and molecular assays.^5^ For comprehensive diagnosis of respiratory infections, a combination of tests is often necessary, including blood culture, sputum microscopy and culture, urinary antigen tests for specific pathogens, and serology.^8^

 As we delve deeper into the various diagnostic approaches for pediatric respiratory infections, it becomes clear that a multi-faceted strategy, incorporating both conventional and molecular methods, is essential for accurate diagnosis and effective treatment. The following sections will explore in detail the specific bacterial pathogens involved in pediatric respiratory infections, the advantages and limitations of different diagnostic techniques, and the promising role of point-of-care testing (POCT) in improving patient outcomes.

## Bacterial Respiratory Infections in Children

 Bacterial respiratory infections are a significant health concern for children, potentially leading to severe complications if left untreated. These infections primarily affect the upper or lower respiratory tract, causing symptoms such as coughing, fever, and difficulty breathing. The most common bacterial pathogens implicated in pediatric respiratory infections include *Streptococcus pneumoniae, Haemophilus influenzae, Moraxella catarrhalis, *and* Staphylococcus aureus*.^9-11^

## Transmission and Risk Factors

 These bacteria are typically transmitted through respiratory droplets expelled during coughing, sneezing, or close contact with infected individuals. Several factors increase the risk of contracting these infections, including: poor hygiene practices, crowded living conditions, weakened immune systems, exposure to environmental pollutants, and inadequate vaccination coverage.^12,13^

 Children, especially those under five years of age, are particularly susceptible due to their developing immune systems and frequent close contact with peers in school or daycare settings.^14,15^

## Clinical Presentation

 The clinical presentation of bacterial respiratory infections in children can vary depending on the pathogen involved and the site of infection. URTIs commonly manifest with symptoms such as: runny or stuffy nose, sore throat, mild cough, and low-grade fever.

 Lower respiratory tract infections (LRTIs), such as bronchitis or pneumonia, often present with more severe symptoms, including: severe cough, chest pain, shortness of breath, high fever, fatigue, loss of appetite, and irritability. In some cases, children may also experience additional symptoms like headaches, body aches, and gastrointestinal disturbances.^16^

## Diagnosis and Treatment

 Accurate diagnosis of bacterial respiratory infections in children is crucial for appropriate treatment and prevention of complications. Healthcare providers typically consider the child’s medical history, physical examination findings, and laboratory tests to make a diagnosis. Laboratory tests may include: blood tests (e.g. complete blood count, C-reactive protein), sputum cultures, chest X-rays, rapid antigen detection tests and molecular diagnostic methods (e.g. PCR).^17-19^

 The choice of diagnostic approach often depends on the severity of symptoms, the child’s age, and the suspected pathogen.^3^

 Treatment of bacterial respiratory infections in children usually involves antibiotic therapy. The selection of antibiotics depends on the type of bacteria suspected or identified and its susceptibility to specific medications. Commonly prescribed antibiotics for pediatric respiratory infections include: amoxicillin, cephalosporins (e.g. cefuroxime, ceftriaxone, etc.), and macrolides (e.g. azithromycin, clarithromycin, etc). It is crucial to follow the prescribed dosage and complete the full course of antibiotics to ensure effective treatment and prevent the development of antibiotic resistance.^20^

## Challenges in Pediatric Respiratory Infection Management

 Despite advances in diagnostic techniques and treatment options, managing bacterial respiratory infections in children presents several challenges:

1. Differentiation between viral and bacterial infections: Many respiratory symptoms can be caused by both viruses and bacteria, making it difficult to determine the need for antibiotic treatment based on clinical presentation alone.^5^
2. Antibiotic resistance: The increasing prevalence of antibiotic-resistant bacteria complicates treatment decisions and emphasizes the need for accurate diagnostic methods to guide appropriate antibiotic use.^4^
3. Sample collection: Obtaining adequate samples for diagnostic testing can be challenging in young children, particularly for lower respiratory tract infections.^7^
4. Rapid diagnosis: The need for quick and accurate diagnosis is crucial in pediatric patients to initiate timely treatment and prevent complications.^21^
5. Overuse of antibiotics: Parental pressure and diagnostic uncertainty often lead to unnecessary antibiotic prescriptions, contributing to the growing problem of antibiotic resistance.^14^

Addressing these challenges requires a multifaceted approach, including improved diagnostic techniques, education of healthcare providers and parents, and the development of rapid, accurate POCT methods. The following sections will delve deeper into the various diagnostic approaches available for pediatric respiratory infections, with a focus on molecular detection methods and POCT.

## Identification and Detection of Bacterial Respiratory Infections in Children

 Accurate and timely identification of bacterial pathogens causing respiratory infections in children is crucial for effective treatment and management. This section explores various detection methods, ranging from conventional techniques to advanced molecular approaches (Table 1).^22,23^

**Table 1:** Comparison of molecular, conventional and serological methods for detection of bacterial infections.

**Method Characteristics**	**Molecular**	**Culture**	**Serological**	**Ref.**
Principle	Amplification and detection	Isolation and growth	Detection of antibodies	^24,25^
Sensitivity	High	Variable	Variable	^26-28^
Specificity	High	High	Variable	
Speed	Rapid	Can take days	Rapid	
Equipment	PCR machine/Amplification kit	Microbiology laboratory setup	ELISA reader/Immunoassay device	^29-31^
Sample	Minimal	Large quantity	Serum or other bodily fluids	^32-34^
Diagnostic purpose	Identifying bacterial strains	Identifying bacterial species	Detecting past or current infection	
Limitations	Requires specialized equipment	May miss slow-growing bacteria	False positives/negatives possible	
Examples of use	PCR, Real-time PCR, LAMP, RPA, NASBA, etc.	Blood agar plate culture	ELISA, Western blot, etc.	^35-37^

###  Conventional Detection Methods

 Conventional methods have long been the cornerstone of bacterial identification in clinical settings. These methods include:

####  Culture-Based Techniques

 Culturing remains a standard approach for identifying bacterial pathogens. However, it has limitations, particularly for fastidious organisms like *Streptococcus pneumoniae*. The sensitivity of culture tests can rapidly diminish after antibiotic administration, potentially leading to false-negative results.^38^ Despite its high specificity, blood culture positivity rates are typically less than 20% in pediatric patients with suspected bacterial infections.^8^

####  Gram Staining

 This rapid and inexpensive method provides initial information about the morphology and gram-reaction of bacteria. However, it lacks specificity and cannot definitively identify the bacterial species.^5^

####  Biochemical Assays

 These tests identify bacteria based on their metabolic characteristics. While useful, they can be time-consuming and may not differentiate between closely related species.^39^

####  Serological Methods

 Serological testing, which detects specific antibodies, can be helpful when conventional methods fail to identify the cause of infection. However, these methods have limitations in patients with compromised immune systems and may not detect acute infections.^40^

###  Advanced Detection Methods

 To address the limitations of conventional techniques, several advanced methods have been developed:

####  Molecular Detection Techniques

 Nucleic acid amplification techniques, particularly PCR, have revolutionized the detection of respiratory pathogens. Multiplex real-time PCR is especially useful, offering rapid diagnosis with universal sensitivity and specificity of 75.9% and 96.5%, respectively.^3^ These methods can detect a very small number of organisms, whether viable or not, making them highly sensitive.^41^

**Figure 1:**
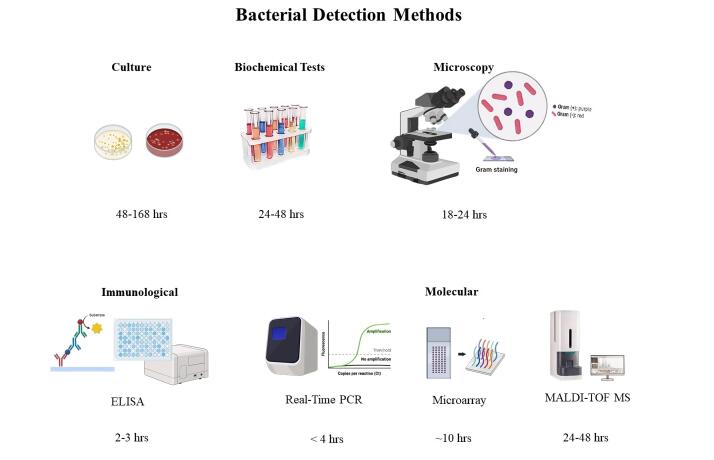
Different Detection Methods Used for Bacterial Infections and their Turnaround Time

 Other molecular methods include:

1. Microarray technologies: These use two-dimensional microchips or three-dimensional beads for simultaneous detection of multiple pathogens.
2. Matrix-assisted laser desorption/ionization time-of-flight mass spectrometry (MALDI-TOF MS): This technique rapidly identifies bacteria based on their protein profiles (Figure 1).^2^

####  Rapid Antigen Detection Tests

 Immunochromatographic urinary antigen tests provide rapid diagnosis for certain pathogens like *Streptococcus pneumoniae* and *Legionella pneumophila*. However, their sensitivity and specificity can be lower in pediatric populations, particularly for pneumococcal infections.^42^

####  Point-of-Care Testing 

 POCT methods are designed to be easy to use, sensitive, and provide rapid results at or near the patient care site. These tests can be broadly categorized into two groups:

1. Tests determining the cause of infection.
2. Tests confirming the presence of specific pathogens.

**Figure 2:**
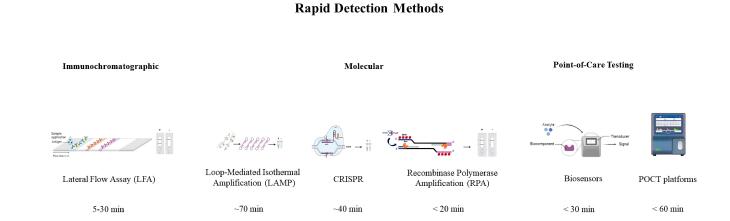
Some Rapid Detection Methods used for Bacterial Infections and their Turnaround Time

For children older than eight years, urine tests for simultaneous identification of *S. pneumoniae* and *L. pneumophila* antigens are commonly used, with a sensitivity of 85%-89% (Figure 2).^16^

 Several PCR-based POCT platforms have been developed that can detect multiple respiratory pathogens within an hour. The Film Array respiratory panel, for instance, has shown promise in reducing hospitalization costs and intravenous antibiotic use.^21^

 Other commercially available respiratory panels include QIAstat-Dx®, Unyvero, DendriChips®, and RespiFinder® SMART 22 FAST. These platforms offer rapid, multiplex detection of various respiratory pathogens.^16^

## Emerging Biomarkers

 Research is ongoing into novel biomarkers for respiratory infections. The C-reactive protein (CRP) test and procalcitonin concentration measurements show promise as POCT tests, although their utility in pediatric populations requires further investigation.^43^

 Other potential biomarkers include:

1. VAPrapid-2: This test measures interleukins IL-1β and IL-8.^44,45^
2. HostDx Sepsis test: This assay examines a large battery of host transcriptome markers in blood for pneumonia diagnosis.^43^

These emerging biomarkers may offer new avenues for rapid and accurate diagnosis of respiratory infections in children, but further clinical trials are needed to validate their effectiveness.^43^

## Challenges and Future Directions

 Despite significant advancements in detection methods, several challenges remain:

1. Sample collection: Obtaining adequate samples, particularly from young children, can be difficult.^46,47^
2. Interpretation of results: The presence of bacterial DNA does not necessarily indicate an active infection, potentially leading to over-diagnosis.^48,49^
3. Cost and accessibility: Advanced molecular techniques may not be readily available in all healthcare settings, particularly in resource-limited areas.^50,51^
4. Rapid detection of antibiotic resistance: There is an ongoing need for methods that can quickly identify antibiotic-resistant strains to guide appropriate treatment.^52^

Future research should focus on developing more accessible, rapid, and comprehensive diagnostic tools that can simultaneously detect pathogens and their antibiotic resistance profiles. Integration of artificial intelligence and machine learning algorithms with diagnostic platforms may further enhance the accuracy and speed of diagnosis.^53,54^

## Antibiotic Resistance in Pediatric Respiratory Infections

 Antibiotic resistance is a growing concern in the management of pediatric respiratory infections. The overuse and misuse of antibiotics have led to the emergence of resistant bacterial strains, complicating treatment and potentially leading to more severe outcomes. This section explores the challenges posed by antibiotic resistance and the methods used to detect resistant strains in pediatric respiratory infections.^55,56^

## Prevalence and Impact of Antibiotic Resistance

 The prevalence of antibiotic-resistant bacteria in pediatric respiratory infections has been increasing globally. A study by Torres et al found that more than 70% of uncertain diagnoses and prescriptions of antibiotics contribute to the rising antibacterial resistance in children.^4^ This trend is particularly concerning for common respiratory pathogens such as *Streptococcus pneumoniae, Haemophilus influenzae, *and* Staphylococcus aureus*.

 The impact of antibiotic resistance in pediatric populations includes: increased morbidity and mortality, prolonged hospital stays, higher healthcare costs, limited treatment options, and potential for spread of resistant strains in communities.^57,58^

## Factors Contributing to Antibiotic Resistance

 Several factors contribute to the development and spread of antibiotic resistance in pediatric respiratory infections:

1. Overuse of antibiotics: Unnecessary prescription of antibiotics for viral infections or minor bacterial infections that could resolve without treatment.^9^
2. Inappropriate antibiotic selection: Use of broad-spectrum antibiotics when narrow-spectrum drugs would suffice.^20^
3. Incomplete treatment courses: Failure to complete the full course of prescribed antibiotics, allowing resistant bacteria to survive and multiply.^16^
4. Antibiotic use in agriculture: The use of antibiotics in livestock can lead to the development of resistant bacteria that may be transmitted to humans.^2^
5. Poor infection control practices: Inadequate hygiene and infection control measures in healthcare settings can facilitate the spread of resistant bacteria.^3^

## Methods to Identify Bacterial Resistance

 Rapid and accurate detection of antibiotic-resistant bacteria is crucial for effective treatment of pediatric respiratory infections. Various methods have been developed to identify resistant strains:

###  Phenotypic Methods

 Traditional phenotypic methods involve culturing bacteria in the presence of antibiotics to determine their susceptibility. These methods include:

1. Disk diffusion test: Measures the zone of inhibition around antibiotic-impregnated disks on agar plates.^59^
2. Broth dilution: Determines the minimum inhibitory concentration (MIC) of antibiotics required to inhibit bacterial growth.^60^
3. Automated systems: Such as VITEK and Phoenix systems, which provide rapid antibiotic susceptibility results.^5,59^

While these methods are widely used, they typically require 24-48 hours to obtain results, which can delay appropriate treatment.^61^

###  Molecular Methods

 Molecular techniques offer faster and more specific detection of antibiotic resistance genes. These methods include:

1. PCR-based assays: Detecting specific resistance genes, such as *mecA* for methicillin resistance in *Staphylococcus aureus*.
2. Microarray technologies: Allowing simultaneous detection of multiple resistance genes.
3. Whole genome sequencing: Provides comprehensive information about resistance genes and other genetic factors contributing to antibiotic resistance.^4^

## Rapid Diagnostic Platforms

 Several rapid diagnostic platforms have been developed to detect antibiotic-resistant bacteria in clinical samples:

1. Verigene® system: This platform can detect resistance genes such as *mecA* and *vanA /B* in gram-positive bacteria, and genes encoding extended-spectrum beta-lactamases (ESBLs) and carbapenemases in gram-negative bacteria.^6,7^
2. FilmArray Blood Culture Identification Panel: This nested PCR-based system can detect resistance genes including *mecA, vanA /B*, and KPC.^5^
3. GeneXpert® system: Offers cartridges for rapid detection of MRSA in various clinical samples.^8^
4. Other platforms: Including NucliSENS EasyQ® KPC, Xpert® Carba-R, eazyplex® SuperBug CRE, and Check-Direct CPE, which target specific resistance mechanisms.^39,41^

###  Emerging Technologies

 New approaches for detecting antibiotic resistance are continually being developed:

1. CRISPR-Cas-based diagnostics: These methods exploit the specificity of CRISPR-Cas systems to detect antibiotic resistance genes rapidly and accurately.^35^
2. Nanopore sequencing: This technology allows real-time sequencing of bacterial genomes, potentially providing comprehensive resistance profiles within hours.^34^
3. Machine learning algorithms: These can predict antibiotic resistance based on genomic data, potentially accelerating the identification of novel resistance mechanisms.^32^

## Challenges and Future Directions

 Despite advances in detection methods, several challenges persist in addressing antibiotic resistance in pediatric respiratory infections:

1. Cost and accessibility: Many advanced detection methods are expensive and not widely available, particularly in resource-limited settings.
2. Turnaround time: While molecular methods are faster than traditional culture-based approaches, there is still a need for even more rapid diagnostics to guide immediate treatment decisions.
3. Complexity of resistance mechanisms: The continual evolution of resistance mechanisms necessitates ongoing updates to detection methods.
4. Interpretation of results: The presence of resistance genes does not always correlate with phenotypic resistance, complicating clinical decision-making.^62,63^

Future research should focus on developing more accessible, rapid, and comprehensive resistance detection methods. Integration of resistance testing into point-of-care devices could significantly improve the management of pediatric respiratory infections. Additionally, efforts to promote antimicrobial stewardship and judicious use of antibiotics in pediatric populations are crucial to mitigate the spread of antibiotic resistance.^22,43^

## Point-of-Care Testing for Pediatric Respiratory Infections

 POCT has emerged as a promising approach for rapid diagnosis of pediatric respiratory infections. These tests offer the potential for faster, more accurate diagnoses, leading to improved patient management and more judicious use of antibiotics. This section explores the various POCT methods available for pediatric respiratory infections, their advantages and limitations, and their potential impact on patient care.^43,64^

## Overview of POCT in Pediatric Respiratory Infections

 POCT refers to diagnostic testing performed at or near the site of patient care. These tests are designed to be user-friendly, provide rapid results, and facilitate immediate clinical decision-making. In the context of pediatric respiratory infections, POCT can be broadly categorized into two main groups: (1) Tests that determine the cause of infection. (2) Tests that confirm the presence of specific pathogens.

 The adoption of POCT in pediatric care has been driven by several factors, including the need for rapid diagnosis, the desire to reduce unnecessary antibiotic use, and the goal of improving overall patient outcomes.^16^

## Types of POCT for Pediatric Respiratory Infections

###  Rapid Antigen Detection Tests

 These tests detect specific antigens from respiratory pathogens in patient samples. Common examples include:

1. Rapid Streptococcus A test: Detects group A streptococcal antigen in throat swabs.
2. Influenza rapid antigen tests: Detect influenza A and B viral antigens in nasopharyngeal samples.
3. RSV rapid antigen tests: Identify respiratory syncytial virus antigens in nasal secretions.

While these tests offer rapid results, their sensitivity can be variable, and negative results often require confirmation with more sensitive methods.^21^

###  Molecular POCT Platforms

 Molecular POCT platforms use nucleic acid amplification techniques to detect pathogens. These methods offer higher sensitivity and specificity compared to antigen-based tests.^65^ Examples include:

1. FilmArray Respiratory Panel (BioFire Diagnostics): This multiplex PCR system can detect multiple respiratory pathogens simultaneously within about an hour. Studies have shown that its use can reduce hospitalization costs and intravenous antibiotic use in pediatric patients.^21^
2. Xpert Xpress (Cepheid): Offers rapid PCR-based detection of influenza and RSV, with results available in less than 30 minutes.^66^
3. ID NOW (Abbott): Provides molecular detection of influenza A & B, RSV, and SARS-CoV-2 in 13 minutes or less.^67^
4. COBAS Liat (Roche): Offers PCR-based detection of multiple respiratory pathogens with a turnaround time of 20 minutes.^68^

These molecular POCT platforms have shown high sensitivity and specificity in pediatric populations, potentially improving diagnostic accuracy and reducing time to appropriate treatment.^69^

###  Isothermal molecular tests to identify bacterial resistance

 The detection and diagnosis of bacterial infections play a crucial role in effective disease management and prevention. Traditional detection methods often involve complex, time-consuming, and expensive processes, hindering their suitability for rapid diagnostics in resource-limited settings. In recent years, isothermal methods have emerged as promising alternatives due to their simplicity, cost-effectiveness, and capability for POCT. This article aims to provide an overview of isothermal methods utilized in the detection of bacterial infections and their potential applications.^9,70,71^

####  A. Loop-Mediated Isothermal Amplification 

 Loop-mediated isothermal amplification (LAMP) is a widely recognized method for the detection of various bacterial infections. By utilizing multiple primers, LAMP allows for rapid amplification of target DNA under isothermal conditions. LAMP offers high specificity, sensitivity, and simplicity, making it suitable for point-of-care diagnostics. For instance, LAMP has been successfully employed for the detection of pathogens such as *Staphylococcus aureus*, *Salmonella* spp., and *Mycobacterium tuberculosis*.^9,16^

####  B. Recombinase Polymerase Amplification 

 Recombinase polymerase amplification (RPA) is another isothermal technique gaining popularity for bacterial infection detection.^72^ RPA utilizes recombinase enzymes and DNA polymerases to amplify target DNA at a constant temperature. RPA is highly specific, fast, and portable, with applications ranging from environmental monitoring to clinical diagnostics. Studies have demonstrated successful utilization of RPA in detecting bacterial pathogens, including *Escherichia coli* and *Klebsiella pneumoniae*.^16,73^

####  C. Nucleic Acid Sequence-Based Amplification 

 Nucleic acid sequence-based amplification (NASBA) is an isothermal amplification technique that enables the detection of RNA-based infections.^74^ NASBA utilizes reverse transcriptase and RNA polymerase to amplify target RNA at a constant temperature. This method offers high specificity and sensitivity, making it suitable for detecting bacterial infections caused by RNA viruses. Notably, NASBA has been employed in the detection of bacterial pathogens such as *Helicobacter pylori* and *Chlamydia trachomatis*.^3,74^

####  D. Recombinase-Aided Amplification 

 Recombinase-aided amplification (RAA) is an isothermal nucleic acid amplification method that relies on recombinase enzymes and DNA polymerases. RAA provides rapid amplification of target DNA or RNA, making it suitable for point-of-care diagnostics. With its simplicity and robustness, RAA shows great potential for detecting bacterial pathogens, including MRSA and *Mycobacterium tuberculosis*.^16,75^

####  E. Whole Genome Amplification 

 Whole genome amplification (WGA) is an isothermal technique used to amplify the entire genome of a bacterial pathogen. By amplifying genomic DNA templates, WGA facilitates downstream molecular analysis and characterization of bacterial infections.^76^ WGA has been applied to the detection of various bacteria, facilitating genomic research and surveillance efforts.^4,77^ Isothermal methods have revolutionized the detection and diagnosis of bacterial infections due to their simplicity, cost-effectiveness, and suitability for POCT. These methods offer rapid, sensitive, and specific results, making them valuable tools for healthcare professionals, researchers, and diagnostic laboratories. As technology advances, isothermal methods hold the potential to further enhance early detection, surveillance, and management strategies for bacterial infections.^78,79^

###  Microfluidic Devices

 Emerging microfluidic technologies are enabling the development of novel POCT devices for respiratory infections. These devices can integrate sample preparation, amplification, and detection into a single, compact system. While many of these technologies are still in development, they show promise for rapid, sensitive, and specific detection of respiratory pathogens in pediatric patients.^80^

###  Host Response Biomarker Tests

 Some POCT methods focus on detecting host response biomarkers to differentiate between viral and bacterial infections. Examples include:

1. CRP POCT: Measures levels of CRP, an acute-phase protein that increases in response to inflammation.^81,82^
2. Procalcitonin POCT: Detects levels of procalcitonin, which tends to be elevated in bacterial infections.^83,84^

While these tests can provide valuable information, their interpretation in pediatric populations can be challenging and often requires consideration alongside other clinical and laboratory findings.^43^

## Advantages of POCT in Pediatric Respiratory Infections

 Implementation of POCT in managing pediatric respiratory infections offers several potential benefits:

1. Rapid results: POCT can provide results within minutes to hours, allowing for faster clinical decision-making.^85,86^
2. Improved antibiotic stewardship: Rapid identification of viral pathogens can reduce unnecessary antibiotic prescriptions.^87,88^
3. Enhanced patient care: Faster diagnosis can lead to more timely and appropriate treatment.^85,89^
4. Reduced healthcare costs: POCT may decrease the need for additional testing and shorten hospital stays.^89,90^
5. Improved infection control: Rapid identification of pathogens can facilitate timely implementation of infection control measures.^91,92^

## Limitations and Challenges of POCT

 Despite its potential benefits, POCT for pediatric respiratory infections faces several challenges;^43,64,89^

1. Cost: Some POCT platforms, particularly molecular-based systems, can be expensive to implement and maintain.^93,94^
2. Quality control: Ensuring consistent test performance across different operators and settings can be challenging.^95,96^
3. Result interpretation: Healthcare providers need proper training to interpret POCT results in the context of clinical presentation.^86,92,97^
4. Limited test menu: Some POCT platforms may not cover all pathogens of interest.^98,99^
5. Regulatory considerations: POCT devices must meet regulatory requirements, which can vary by region.^92,100^

## Impact on Patient Care and Antibiotic Stewardship

 The implementation of POCT in pediatric respiratory infections has shown promising results in improving patient care and promoting antibiotic stewardship. A randomized clinical trial by Mattila et al found that the use of POCT for respiratory pathogens significantly reduced antibiotic use in children without compromising patient outcomes.^16^

 Moreover, rapid identification of viral pathogens through POCT can help reassure parents and clinicians, potentially reducing unnecessary antibiotic prescriptions and follow-up visits. This approach aligns with global efforts to combat antibiotic resistance by promoting more judicious use of antimicrobial agents in pediatric populations.^9^

## Future Directions

 The field of POCT for pediatric respiratory infections continues to evolve rapidly.^64,101^ Future developments may include:

1. Integration of artificial intelligence: Machine learning algorithms could enhance the interpretation of POCT results, potentially improving diagnostic accuracy.^102,103^
2. Multiplexed detection of pathogens and antibiotic resistance: Next-generation POCT devices may simultaneously identify pathogens and their antibiotic resistance profiles.^53,54^
3. Wearable and smartphone-connected devices: These could enable continuous monitoring of respiratory parameters and facilitate remote diagnosis.^104,105^
4. Improved sample collection methods: Development of less invasive and more child-friendly sampling techniques could enhance the acceptability of POCT in pediatric populations.^106,107^

## Conclusion

 Molecular testing has significantly improved respiratory pathogen detection and is now regarded as the new “gold standard.” Although these tests have grown in popularity, criteria such as patient demographics (adult, pediatric, and immunocompromised), laboratory size, testing purpose (regular or urgent care), and cost-benefit ratio should be addressed before implementing a specific assay. Molecular diagnostics offer high sensitivity and the potential for timely delivery of actionable information, which can reduce diagnostic uncertainty and help inform early treatment decisions more effectively than conventional culture and antigen-based methods. Decisions for the deployment of molecular diagnostics in the hospital laboratory and for the hospital system should take into account their use in guiding procedures and policies, such as in-hospital epidemiology and antibiotic stewardship. As technology advances and data-supporting best practices emerge, policies for successful utilization must be assessed on a constant basis. Future research will be required to demonstrate the clinical efficacy of current and future tests in a variety of patient demographics and resource-constrained situations.
